# Influence of Fly Ash Content on the Durability of Mortar Specimens under Dry/Wet Sulfate Attack

**DOI:** 10.3390/ma17010113

**Published:** 2023-12-25

**Authors:** Yage Zhang, Dongge Wu, Yushan Wang, Yang Zhou, Shan Wang, Yi Zhao

**Affiliations:** 1 College of Water Conservancy & Architectural Engineering, Shihezi University, Shihezi 832000, China; zygnlzc@163.com (Y.Z.); zy0415154@shzu.edu.cn (Y.Z.); 2 Xinjiang Hongyuan Construction Group Co., Ltd., Cocodala 835219, China; wdg--yn@126.com; 3 Xinjiang Tianshan Cement Co., Ltd., Urumqi 830000, China; 4 China Construction Third Engineering Bureau Group Co., Ltd., Nanjing 210000, China; wangshansd@foxmail.com

**Keywords:** fly ash, steam curing, dry/wet sulfate, strength evolution model, erosion mechanism

## Abstract

To investigate the durability of cementitious materials under complex environmental conditions in Xinjiang, this study conducted durability tests on mortar specimens with different fly ash contents under dry/wet sulfate attack conditions, with standard curing and steam curing at 70 °C. The appearance loss and flexural and compressive strength variations in the specimens were analyzed, and an evolution model of the mortar strength under a dry/wet sulfate attack was established. Moreover, XRD and SEM techniques were used to characterize the erosion products and microstructure, and to explore the erosion resistance mechanism of fly ash cementitious materials. The results showed that, after 160 cycles of erosion, the flexural strength of the specimens decreased with the increase in the fly ash content. In the context of steam-cured mortar specimens, throughout the entire erosion period, specimens with a fly ash content of 45% exhibited the highest relative compressive strength. The established strength evolution model had a minimum determination coefficient of 0.879, indicating a good agreement between the model and experimental results. Microscopic research showed that fly ash would undergo a pozzolanic reaction under the action of sulfate and calcium hydroxide, which was beneficial to the improvement of the erosion resistance. As the fly ash content increased, the erosion products of the specimens gradually became dominated by gypsum.

## 1. Introduction

As the global economy continues to grow, the construction of high-speed railways is gradually gaining momentum worldwide. As an essential transportation hub for the development of the “Belt and Road” initiative, the construction of high-speed railways in the Xinjiang region has become a necessary measure. To shorten the construction period, reduce environmental pollution, simplify the construction process, and lower costs, steam-cured concrete prefabricated components, such as sleepers, track slabs, and prestressed simply supported beams, are primarily used [1,2]. Although steam curing accelerates the hydration of cement components in prefabricated parts, coarsens the pore structure of the concrete, and increases the permeability of the concrete [3,4], in the Xinjiang region of northwestern China, the unique topography and climatic variations between the northern and southern parts contribute to variations in the composition and content of salts in the soil. Notably, this region hosts extensive saline–alkali soils containing sulfate ions. The sulfate ions can be adsorbed and enriched in concrete materials through dry–wet cycles and fine adsorption processes [5,6]. The durability of steam-cured cementitious materials in service under complex environmental conditions is comparatively compromised. Considering the sulfate ion concentration associated with sulfate attack in the Xinjiang region, this study selected a confidence interval of 85% for the SO_4_^2−^ ion concentration, specifically 20,250 mg/L, for dry–wet sulfate attack testing. Therefore, improving the service quality of steam-cured prefabricated components in complex environments is an urgent issue to be addressed.

China, being a major consumer of coal resources globally, generates a substantial proportion of solid waste, with fly ash constituting the largest share after coal combustion [7]. Addressing the question of how to appropriately manage this solid pollutant, fly ash, holds significant importance in transforming waste into a valuable resource and aligning with the environmental sustainability principles of a “Green China”. Sun Daosheng et al. [8,9,10] conducted erosion experiments on standard-cured cement-based materials with different fly ash cementitious materials, semi-immersed in a 5% (mass fraction) sodium sulfate solution. The study showed that increasing the amount of fly ash increased the proportion of capillary pores in the mortar, resulting in a significant increase in the capillary absorption and transport characteristics in the water and sodium sulfate solution, increasing the concentration of sulfate ions in the salt crystallization zone of the mortar specimens, causing more severe physical salt crystallization erosion damage. Wei Yimeng et al. [11,12] conducted experiments to investigate the impact of high-volume fly ash (FA) on the durability of sulfate attack under dry–wet cycling conditions in cement-based materials. The research findings indicated that the inclusion of a high volume of fly ash did not enhance the resistance of concrete to sulfate attack; instead, it led to a decrease in the matrix strength, adversely affecting the durability of the concrete. It is evident that, under standard-curing conditions, a high content of fly ash diminishes the resistance of Portland-cement-based materials to dry–wet sulfate attack. Ming-fang Ba et al. [13,14] analyzed the effect of fly ash on the chloride ion penetration resistance of steam-cured concrete. The study showed that incorporating fly ash can improve the late strength of steam-cured concrete, reduce crack sensitivity, enhance the resistance of steam-cured concrete to chloride ion penetration, and improve the durability of the concrete. Wu Kegang et al. [15,16,17,18,19] investigated the impact of high-volume fly ash content on the porosity, including both the macro- and micropores, of steam-cured fly ash concrete. Their study revealed that, under steam-curing conditions, the porosity, including both large and small capillary pores, of steam-cured fly ash concrete was lower than that of standard-cured fly ash concrete when the fly ash content exceeded 40%. This research suggests that steam-curing conditions can enhance the impermeability of fly ash cementitious materials, optimize their pore structure, and improve their resistance to erosion. Nevertheless, systematic investigations into the durability of fly ash cementitious materials from Xinjiang under dry–wet sulfate attack conditions remain limited.

This study aims to investigate the durability of cementitious materials in the complex environmental conditions of Xinjiang, China. Employing P·Ⅰ 42.5-grade cement, our focus centers on analyzing the impact of varying fly ash contents and different curing regimes on the visual appearance and flexural and compressive strength of fly ash mortar specimens. At the same time, a mortar strength evolution model under wet–dry and sulfate action is established, and XRD, SEM, and other means are used to characterize the erosion products and microstructures. This will help explore the damage mechanism of steam-cured fly ash cement mortar specimens under erosion, and provide a theoretical basis and technical support for the production and engineering application of steam-cured fly ash cement-based materials in the corrosive environment of Xinjiang.

## 2. Experimental Design

### 2.1. Material Properties

**Figure 1:**
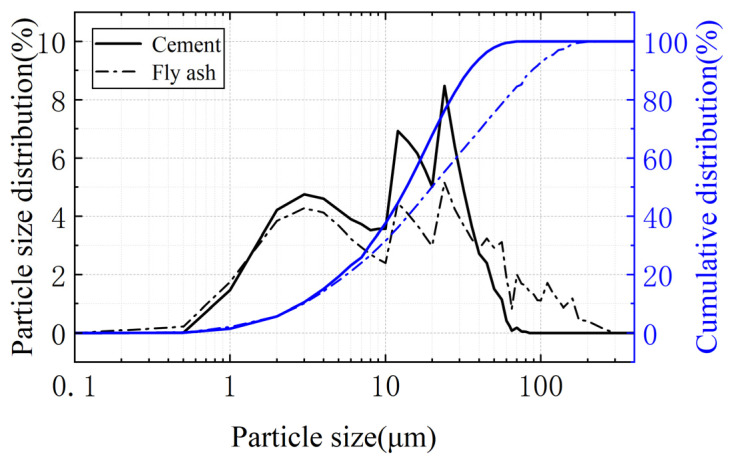
The size distribution chart of the cement and fly ash.

The P·Ⅰ 42.5-grade cement used in this study was produced by Xinjiang Tianshan Cement Co., Ltd. Dabancheng Branch, China, with a specific surface area of 329.3 m^2^/kg. The chemical composition is shown in Table 1, and the cement particle size distribution curve and cumulative distribution curve of the cement particles are shown in Figure 1. The fly ash provided by Xinjiang Tianshan Cement Co., Ltd., Dabancheng Branch, has an as-received specific surface area of 303.6 m^2^/kg and a median particle size of 32.03 μm. Its chemical composition is shown in Table 1, while the fly ash particle size distribution curve and cumulative distribution curve of cement particles are shown in Figure 1. The sand employed in this study was sourced from Xiamen ACE Standard Sand Co., Ltd., a producer of Chinese ISO standard sand. It possesses a fineness modulus of 3.0, and its particle size distribution spans from 0.08 mm to 2 mm. Tap water from Dabancheng was used as the water source.

### 2.2. Material Mixes and Maintenance Systems

The fly ash content was set at 0%, 15%, 30%, and 45% of the total binder material, with a water-to-cement ratio of 0.5 and a binder-to-sand ratio of 1:3 for the mortar specimens, which have dimensions of 40 mm × 40 mm × 160 mm [20]. The mortar specimens were cured at a temperature of (20 ± 1) °C and a relative humidity above 90% for 24 h. After labeling and demolding the specimens, the standard-cured specimens were placed in water at a temperature of (20 ± 1) °C, while the steam-cured specimens were placed in a steam curing chamber at 70 °C for 12 h (0.5 h of standing, 2.5 h of heating, 6 h of heat preservation, and 3 h of cooling). After cooling, the specimens were placed in water at a temperature of (20 ± 1) °C for curing.

### 2.3. Test Method

After curing the specimens for 28 days, standard-cured and steam-cured specimens were removed from the water, dried on the surface, and placed in a sulfate wet–dry test chamber. Based on the sulfate ion concentration for sulfate attack in Xinjiang region reported in the literature [21], a sulfate ion concentration of 20,250 mg/L at an 85% confidence interval for the noncultivated land was selected as the sulfate ion concentration for the erosion solution. The solution prepared with deionized water was added to the storage tank, and the following settings were applied: 15 h of immersion, 1 h of air-drying, an (80 ± 5) °C drying temperature, 6 h of drying time, a (25 ± 5) °C cooling temperature, and 2 h of cooling time. After completing 25, 50, 80, 110, and 160 wet–dry sulfate-attack cycles, the flexural and compressive strength of the specimens were measured, and the appearance changes were recorded. The experiment was terminated when the compressive strength corrosion coefficient according to GB/T50082-2009 [22] was less than 75%, or the number of wet–dry cycles reached the preset number.

### 2.4. Testing Instruments

In accordance with the national standard GB/T 17671-2021 “Cement Mortar Strength Test Method (ISO Method)” [20], the compressive and flexural strength of all mortar specimens were tested. The flexural strength and compressive strength of the mortar were tested using a Wuxi Jianyi DKZ-6000 electric flexural testing machine and a Shenzhen SanSi UTM 7305 compressive–flexural integrated testing machine, respectively. After vacuum-drying the specimens, they were crushed in a mortar, ground to a fine powder, sieved through a 200-mesh screen, and sampled. After undergoing vacuum drying, the specimens were crushed and ground using a mortar and pestle, followed by sieving through a 200-mesh screen for sampling. Subsequently, phase analysis of the specimens was conducted using a German Bruker D8 Advance X-ray diffractometer (XRD). Simultaneously, the morphology of the erosion products on the specimen surface, reaching a depth of 2 mm, was observed using a field-emission scanning electron microscope (JSM-7610F Plus).

## 3. Test Results and Analysis

### 3.1. Appearance Changes

**Figure 2:**
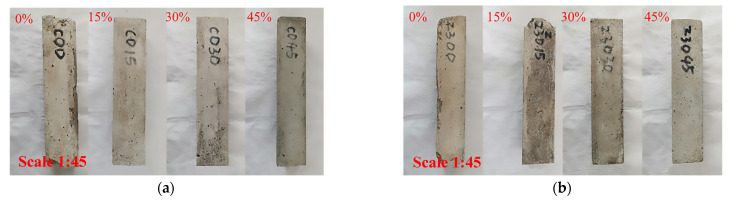
The appearance of the mortar specimens after 160 cycles of erosion. (**a**) Standard curing, (**b**) Steam curing at 70 °C.

As shown in Figure 2, with the increase in the fly ash content, the integrity of the specimens improved, and the cracks and mortar spalling decreased. White crystal precipitates were observed on the surface of all specimens. In comparison to the specimens under standard-curing conditions, the specimens cured at 70 °C showed corner loss and exposed-edge aggregate when the fly ash content was 15%. Although there was overall fracturing, no large cracks appeared, which is consistent with the appearance characteristics of gypsum as the primary erosion product. These results indicate that, under the steam curing at 70 °C, the integrity of specimens improved, with an increase in the fly ash content. Under standard-curing conditions, specimens with 15% or 0% fly ash content exhibited better erosion resistance. It is speculated that, in the solution with an SO_4_^2−^ concentration of 20,250 mg/L, the sulfate erosion products were primarily gypsum.

### 3.2. Mechanical Properties

#### 3.2.1. Flexural Strength

**Figure 3:**
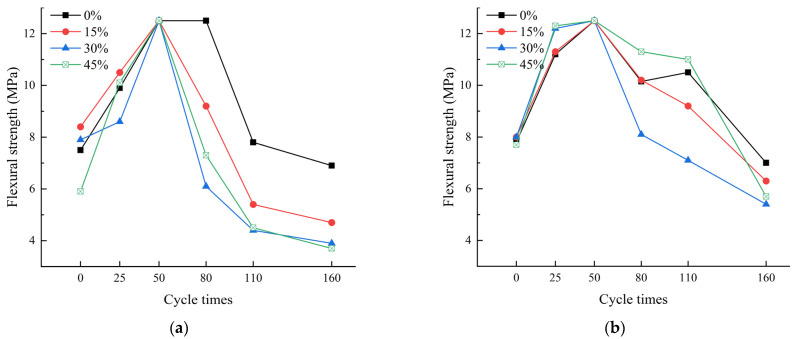
Flexural strength of specimens. (**a**) Standard curing, (**b**) Steam curing at 70 °C.

As shown in Figure 3a, under the standard-curing condition of 20 °C, with the extension of the erosion cycles, the flexural strength of all specimens showed an increasing trend first, and then decreased. Without erosion, the 28-day flexural strength of the specimens with 15%, 30%, and 45% fly ash content were 112.00%, 105.33%, and 78.67% of the 7.5 MPa flexural strength of the pure cement mortar specimens, respectively. The incorporation of an appropriate amount of fly ash is indicated to be advantageous for the 28-day flexural strength of the specimens. This was attributed to the fine particles of the fly ash, which filled the voids in the concrete, consequently resulting in an enhancement of the 28-day flexural strength [23]. But when the fly ash content increased to 45%, it was detrimental to the 28-day flexural strength of the specimens. After 50 erosion cycles, the flexural strength of all specimens reached the maximum. However, after 50 erosion cycles, the flexural strength of the remaining specimens gradually decreased, except for the pure cement mortar specimens, which reached a flexural strength of 12.5 MPa after 80 erosion cycles. After 160 erosion cycles, the flexural strength of the pure cement mortar specimens was 6.9 MPa, while the flexural strength of the specimens with 15%, 30%, and 45% fly ash content were 68.12%, 56.52%, and 53.62% of the pure cement mortar specimens, respectively. This suggests that, under standard-curing conditions, the increase in the fly ash content was detrimental to the sulfate erosion resistance of the specimens.

As shown in Figure 3b, under the steam-curing condition of 70 °C, the flexural strength of all specimens showed an increasing trend at first, and then decreased with the extension of the erosion cycles. Without the erosion, the 28-day flexural strength of the specimens with 15%, 30%, and 45% fly ash content were 102.56%, 102.56%, and 98.72% of the 7.8 MPa flexural strength of the pure cement mortar specimens, respectively. This indicates a minor influence of the increased fly ash content on the 28-day flexural strength. However, under steam curing at 70 °C, the mortar specimens with a lower fly ash content could, to some extent, stimulate the reactivity of fly ash volcanic ash, enhancing the 28-day flexural strength of the fly ash specimens [24]. After 50 erosion cycles, the flexural strength of all specimens reached the maximum value of 12.5 MPa; after 160 erosion cycles, the flexural strength of the specimens with 0% fly ash content decreased to 7 MPa, while the flexural strength of the specimens with 15%, 30%, and 45% fly ash content were 90.00%, 77.14%, and 81.43% of the pure cement mortar specimens, respectively. In conclusion, under the steam-curing condition of 70 °C, the pure cement mortar specimens exhibited good erosion resistance.

The results indicate that, regardless of the curing method employed, fly ash is not suitable for long-term use. However, steam curing can lead to improved flexural strength in the fly ash mortar specimens. Under standard-curing conditions, when the fly ash content was 15% and 30%, the 28-day flexural strength of the specimens increased; however, when the fly ash content increased to 45%, the 28-day flexural strength of the standard-curing specimens decreased, while the impact on the 28-day flexural strength of the steam-cured specimens at 70 °C was not significant. After 50 cycles of erosion, the flexural strength of the standard-cured fly ash mortar specimens was consistently lower than that of the steam-curing cement mortar specimens. Similarly, for the steam-cured mortar specimens with a 15% fly ash content and a 45% fly ash content, the flexural strength was lower than that of the pure cement mortar specimens after 80 and 125 erosion cycles, respectively. After 160 cycles of erosion, the flexural strength of all specimens was found to be lower than that of the pure cement mortar specimens. Additionally, the flexural strength of the specimens decreased with an increase in the dosage of fly ash. Especially under standard-curing conditions, where the increase in fly ash content led to a significant decrease in flexural strength. Furthermore, there was no significant difference in the flexural strength of the pure cement mortar specimens after 160 erosion cycles between the steam-curing at 70 °C and standard-curing conditions.

#### 3.2.2. Compressive Strength

**Figure 4:**
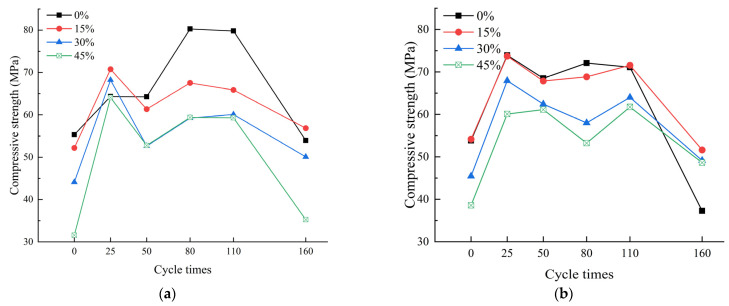
Compressive strength of specimens. (**a**) Standard curing, (**b**) Steam curing at 70 °C.

Figure 4a demonstrates that, under the standard-curing conditions, throughout the observed erosion cycles, the compressive strength of the mortar specimens exhibited an overall trend of increasing at first and then decreasing with the rise in the number of erosion cycles. The specimens containing fly ash showed two peaks in the compressive strength. This is due to the continued hydration of the fly ash during the erosion phase, where the hydration products filled the internal voids of the mortar specimens, thereby enhancing the compressive strength. Simultaneously, the incorporation of the fly ash effectively resisted the later-stage strength degradation of the cementitious material induced by the steam curing [23]. Before the erosion, the 28-day compressive strengths of the specimens with fly ash contents of 15%, 30%, and 45% were 94.31%, 79.71%, and 57.08% of the pure cement mortar specimens’ 55.34 MPa, respectively. This indicates that the increase in fly ash content was detrimental to the 28-day compressive strength of the specimens [25], but the negative impact was relatively small when the fly ash content was 15%. The compressive strength of the pure cement mortar specimens reached its maximum value after 80 erosion cycles, while the specimens containing fly ash reached their maximum value after 25 erosion cycles. The specimens with a 15% fly ash content reached their peak after 80 erosion cycles, while those with 30% and 45% fly ash contents reached their peak after 110 erosion cycles. After 160 erosion cycles, the compressive strength of the pure cement mortar specimens was 53.95 MPa, while the specimens with fly ash contents of 15%, 30%, and 45% had compressive strengths of 105.39%, 92.81%, and 65.36% of the pure cement mortar specimens, respectively. The specimens with a 15% fly ash content showed better erosion resistance than the pure cement specimens.

Figure 4b demonstrates that, under the steam-curing conditions, the overall trend of the development of the compressive strength in the observed erosion cycles was to increase at first and then decrease. Before the erosion, the 28-day compressive strengths of the specimens with fly ash contents of 15%, 30%, and 45% were 100.58%, 84.42%, and 71.66% of the pure cement mortar specimens’ 53.84 MPa, respectively. This indicates that the increase in the fly ash content was detrimental to the 28-day compressive strength of the specimens, but the negative impact was relatively small when the fly ash content was 15%. From 0 to 110 erosion cycles, the compressive strength of the specimens with 0% fly ash content formed the upper-envelope line for all specimens, and the compressive strength decreased with the increase in the fly ash content. After 160 cycles, the compressive strengths of the specimens with fly ash contents of 15%, 30%, and 45% were 51.62 MPa, 49.14 MPa, and 48.62 MPa, which was 138.48%, 131.83%, and 130.45% of the standard-cured specimens’ 37.27 MPa, respectively. Therefore, the erosion resistance of specimens containing fly ash was better under steam curing at 70 °C, and the difference in compressive strength was relatively small.

In summary, under the standard-curing conditions, specimens with 15% fly ash content showed better sulfate erosion resistance. Under steam curing at 70 °C, specimens containing fly ash were more resistant to erosion, and after 160 cycles of erosion, the specimens with varying percentages of fly ash exhibited minimal differences in compressive strength. Furthermore, post-160 cycles of erosion, the compressive strength of the steam curing at 70 °C of the pure cement mortar specimens showed a slight decrease compared to the standard-cured pure cement mortar specimens. The steam curing at 70 °C had a detrimental effect on the compressive strength of the specimens.

#### 3.2.3. Compressive Strength Evolution Model

(1) Model building

After the erosion process, the loss of compressive strength in most specimens did not reach 25%. Therefore, in order to determine the number of erosion cycles required for the compressive strength loss to reach 75% of the initial compressive strength, a mortar strength prediction model was established in this section [26,27]. This model uses a relative compressive strength coefficient to characterize the degree of change in the compressive strength of the mortar specimens f(x,n). The expression is:

(1) $$f(x,n)=\frac{A_{\left(x,n\right)}}{A_{\left(x,0\right)}}\times 100\%$$

where $A_{\left(x,n\right)}$ represents the compressive strength of the mortar specimens with a fly ash content of x% after n cycles of dry–wet cycle erosion; $A_{\left(x,0\right)}$ represents the compressive strength of the mortar specimens with a fly ash content of x% and unexposed to erosion; $A_{\left(x,n\right)}$ has a good polynomial relationship with the fly ash content of x% and the number of wet–dry erosion cycles n. Based on the principle of bivariate polynomial fitting and 48 experimental sample data, a mortar relative strength prediction model was established:

(2) $$\begin{array}{ll} f(x,n) & =(B+Cx+Dn+Ex^{2}+Fxn+Gn^{2}+Hx^{3}+Ix^{2}n+Jxn^{2}+\\ & Kn^{3}+Lx^{4}+Mx^{3}n+Nx^{2}n^{2}+Oxn^{3}+Pn^{4})/A_{\left(x,0\right)} \end{array}$$

The variables in this equation include the fly ash content, which is x%, and the number of erosion cycles, n; g(x,n) is the measured value of the relative compressive strength; f(x,n) is the relative compressive strength function that needs to be fitted so that it satisfies n = 0, 25, 50, 80, 110, 160:

(3) $$\Delta ={\sum \left[f(x,n)-g(x,n)\right]}^{2}\to 0$$

The fitting formulas for the relative compressive strength of the mortar specimens under standard-curing and 70 °C steam-curing conditions are obtained as follows:

Standard curing:

(4) $$\begin{array}{ll} f(x,n) & =(93.54060540+0.01695525x+2.57812757n+0.13846492x^{2}-\\ & 0.04212602xn-0.07176795n^{2}-0.00714485x^{3}+0.00237397x^{2}n-\\ & 0.00018469xn^{2}+0.00077963n^{3}+0.00009342x^{4}-0.00000422x^{3}n-\\ & 0.00001313x^{2}n^{2}+0.00000268xn^{3}-0.00000269n^{4})\times 10^{-2} \end{array}$$

Steam curing:

(5) $$\begin{array}{ll} f(x,n) & =(99.69280764-0.00315059x+3.15812831n-0.02571347x^{2}+\\ & 0.00291232xn-0.08650627n^{2}+0.00176050x^{3}+0.00011797x^{2}n-\\ & 0.00018003xn^{2}+0.00086803n^{3}-0.00002629x^{4}+0.00000817x^{3}n-\\ & 0.00000445x^{2}n^{2}+0.00000172xn^{3}-0.00000286n^{4})\times 10^{-2} \end{array}$$

**Figure 5:**
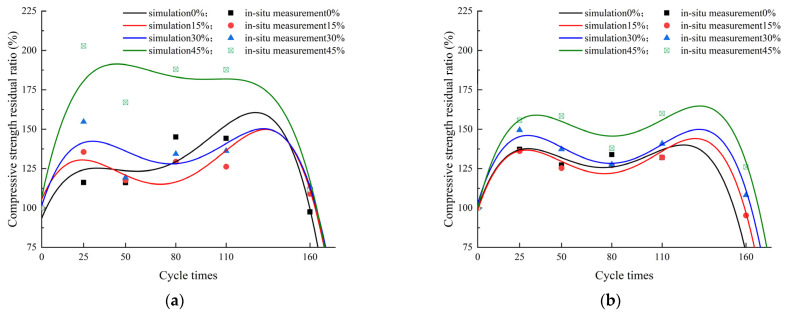
Simulation diagram of the relative compressive strength by erosion. (**a**) Standard curing, (**b**) Steam curing at 70 °C.

Based on Figure 5, it can be observed that the relative compressive strength loss rate of the specimens with different fly ash contents varies, and the number of erosion cycles at which the compressive strength loss reaches 25% also slightly differs. Under the standard-curing conditions, Figure 5a illustrates that the specimens containing 45% fly ash exhibited the highest relative compressive strength after undergoing 0 to 160 cycles of erosion. The optimal performance in resisting sulfate attack was observed in these specimens. The specimen with the 15% fly ash content required the most erosion cycles to reach a relative compressive strength loss of 75%. Under the condition of 70 °C steam curing, Figure 5b illustrates that the specimens containing 30% and 45% fly ash were more suitable to erosion under the conditions of 70 °C steam curing. Additionally, under the same number of erosion cycles, the specimens with 45% fly ash exhibited the highest relative compressive strength. The specimens with 45% fly ash endured the most erosion cycles before reaching a 75% loss in the relative compressive strength. Furthermore, with the increase in the erosion cycles, the enhancing effect of the fly ash gradually became apparent, which could delay the rate and process of the compressive strength deterioration in the concrete.

(2) Model evaluation

To validate the rationality of the relative compressive strength coefficient model of the mortar specimens under the dry–wet sulfate action, four evaluation indicators, including the sum of squares of errors (SSEs), the mean square error (MSE), the root mean square error (RMSE), and the determination coefficient (R-square), were used to evaluate the fitting model with experimental measured values.

Table 2 shows that, under the dry–wet sulfate erosion effect, the maximum values of the variance, mean square error, and root mean square error for the mortar specimens with the different fly ash contents were 2.512 × 10^−1^, 1.047 × 10^−2^, and 1.023 × 10^−1^, respectively, while the minimum value of the determination coefficient was 0.879. Therefore, it can be concluded that the mathematical model established by this polynomial regression has a high feasibility, as the relative compressive strength change trend obtained from mathematical simulation is consistent with the experimental results, indicating a good agreement between the two.

### 3.3. Microanalysis

#### 3.3.1. XRD

**Figure 6:**
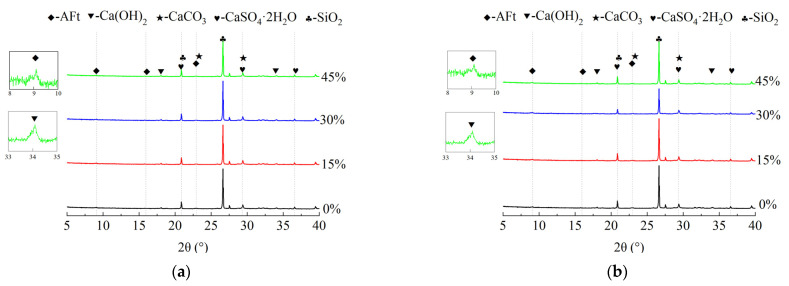
XRD of mortar products after 80 cycles of erosion. (**a**) Standard curing, (**b**) Steam curing at 70 °C.

Based on the XRD results shown in Figure 6a,b, the main phase components of the mortar specimens were identified as SiO_2_, Ca(OH)_2_, CaCO_3_, AFt, and CaSO_4_·2H_2_O [28]. Simultaneously, it can be observed that there is no significant variation in the types of substances formed on the specimens after the erosion under different levels of fly ash content. SiO_2_ is a component present in standard sand, and the weak diffraction peak of Ca(OH)_2_ is due to the reaction of low-concentration sulfate with Ca(OH)_2_ and hydrated calcium aluminate to form ettringite. When the sulfate concentration exceeds 1000 mg/L, gypsum will be generated by the reaction between sulfate and Ca(OH)_2_. In addition, because the dry–wet cycle test used a drying method, Ca(OH)_2_ will gradually carbonate to form CaCO_3_ in the later stage of erosion, which will also lead to a decrease in the intensity of the Ca(OH)_2_ diffraction peak.

#### 3.3.2. SEM

**Figure 7:**
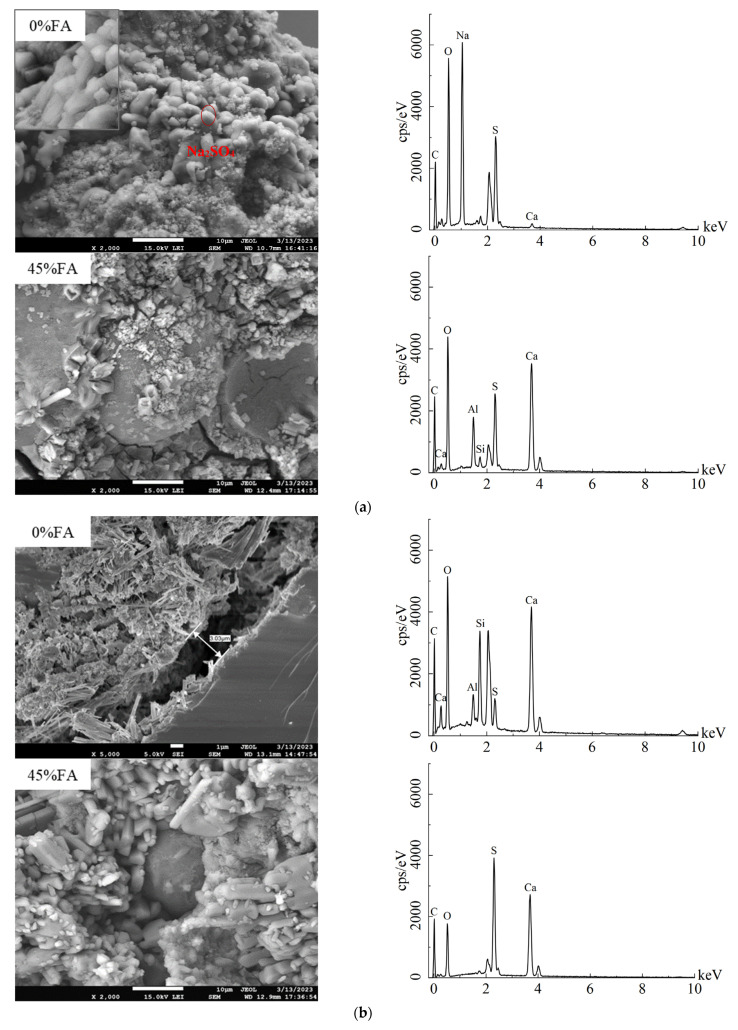
SEM of the mortar corrosion products after 160 cycles. (**a**) Standard curing, (**b**) Steam curing at 70 °C.

According to the results of Figure 7a, under the standard-curing conditions, when the fly ash content was 0%, the integrity of the internal structure of the mortar deteriorated after 160 cycles of erosion, with sodium sulfate crystals being the main crystalline product and carbonation being a secondary phenomenon. This is because the pure cement specimen was relatively dense, and the sulfate solution was not easily permeable to the pores. Therefore, under the combined effects of sulfate crystallization and wet–dry cycles, the specimen will crack and gradually be carbonated to form calcium carbonate. These substances adhere to the pores of the mortar, causing the pores to develop into elongated cracks and fissures, eventually leading to mortar specimen failure. This is consistent with the appearance of the specimen’s failure and strength development. When the fly ash content was 45%, the fly ash participated in the reaction and attached to the surface of the erosion product. Calcium aluminate crystals in the shape of needles or rods, and gypsum in the form of plates, grow from the pore walls and crack edges to the center, but the distribution of the erosion products is uneven.

Based on the results in Figure 7b, it can be seen that, under the steam-curing conditions, the main erosion products in the cracks of the pure cement specimen after 160 cycles of erosion were ettringite and gypsum, which grow in the interface transition zone. Meanwhile, under the action of wet–dry cycles, cracks were formed in the specimen and gradually carbonated. However, when the fly ash content was 45%, the density of the hydration products increased, and the number of cracks was relatively few compared to the specimen without the fly ash. Needle-like ettringite and plate-like gypsum mainly grow on the surface of the fly ash, which has a rough surface with etched marks, and the connection between the fly ash and the hydration products of the cement is close.

In summary, for the specimens under standard curing, the antierosion strength was higher with the 0% fly ash content, and the main erosion product was sodium sulfate crystal. With the increase in the fly ash content, the main erosion product gradually changed to gypsum. As for the specimens under steam curing, with the increase in the fly ash content, the erosion products were mainly gypsum and ettringite, and gradually transformed into gypsum erosion. Additionally, for the specimen with the 45% fly ash content under steam curing at 70 °C, the amount of cement hydration products and secondary fly ash hydration products was increased, the pores were optimized, and the fly ash continued to undergo a pozzolanic reaction in the sulfate environment, which is beneficial to the improvement of the antierosion strength. This is consistent with the two rising stages of the compressive strength of the specimens with the fly ash content.

## 4. Discussion

1. This study investigated the durability of steam-cured high-volume fly ash cementitious materials under dry–wet sulfate attack conditions with a sulfate ion concentration of 20,250 mg/L. Further extensions of the dry–wet sulfate attack investigations at different sulfate ion concentrations should be considered.
2. The present study focused solely on the influence of two factors, namely, the fly ash content and the curing regimes, on the basic mechanical properties and durability of the fly ash cement mortar specimens. Other factors, such as the constant temperature duration and dwell time, have not been explored and could be addressed in subsequent research.

## 5. Conclusions

In this study, the influence of the fly ash content on the durability of the mortar specimens under dry–wet sulfate exposure was investigated. The following conclusions can be drawn:

1. The minimum correlation coefficient between the mathematical simulation of the relative compressive strength and the actual experimental results was 0.879, indicating that the model is reliable. Predictive modeling was employed to assess the specimens with a relative compressive strength of 75%. It was observed that the specimens with the 15% fly ash content under the standard-curing conditions and the specimens with the 45% fly ash content under the steam-curing conditions exhibited favorable resistance to the sulfuric acid attack.
2. The pure cement mortar specimens exhibited a single stage of compressive strength growth, while the mortar specimens with the fly ash content exhibited two stages of compressive strength growth. This phenomenon was attributed to the continued hydration of the fly ash during the erosion phase, resulting in an increase in the compressive strength. Simultaneously, the incorporation of the fly ash proved effective in mitigating the late-stage strength degradation of the cementitious matrix induced by steam curing.
3. Under standard-curing conditions, with the increase in the fly ash content, the main sulfate erosion products gradually changed from sodium sulfate to ettringite and gypsum. However, under the steam-cured condition at 70 °C, the increase in the fly ash content led to a change in the main sulfate erosion products from gypsum and ettringite to mainly gypsum.

## Figures and Tables

**Table 1:** Chemical composition of the cement and fly ash (wt.%).

Component	${SiO}_{2}$	${Al}_{2}O_{3}$	${Fe}_{2}O_{3}$	CaO	MgO	$K_{2}O$	${Na}_{2}O$
Cement/%	22.46	4.72	3.63	62.42	1.28	0.4	0.22
Fly ash/%	54.69	20.17	6.24	8.24	2.46	1.64	1.14

**Table 2:** Evaluation index of the relative compressive strength coefficient model.

Curing Conditions	SSE/10-2	MSE/10-3	RMSE/10-2	R-Square
Standard curing	25.12	10.47	10.23	0.879
Steam curing	2.84	1.18	3.44	0.976

## Data Availability

Data are contained within the article.
